# Implementation of Model-Based Dose Adjustment of Tobramycin in Adult Patients with Cystic Fibrosis

**DOI:** 10.3390/pharmaceutics14081750

**Published:** 2022-08-22

**Authors:** Jérémy Reverchon, Vianney Tuloup, Romain Garreau, Viviane Nave, Sabine Cohen, Philippe Reix, Stéphane Durupt, Raphaele Nove-Josserand, Isabelle Durieu, Quitterie Reynaud, Laurent Bourguignon, Sandrine Charles, Sylvain Goutelle

**Affiliations:** 1 Hospices Civils de Lyon, GH Nord, Service de Pharmacie, 69004 Lyon, France; 2 Univ Lyon, Université Claude Bernard Lyon 1, UMR CNRS 5558, LBBE—Laboratoire de Biométrie et Biologie Évolutive, 69622 Villeurbanne, France; 3 Hospices Civils de Lyon, Pharmacie Centrale, 69230 St. Genis Laval, France; 4 Hospices Civils de Lyon, Groupement Hospitalier Sud, Laboratoire de Pharmaco-Toxicologie, 69495 Pierre-Bénite, France; 5 Hospices Civils de Lyon, Centre de Ressources et de Compétences de la Mucoviscidose, 69500 Bron, France; 6 Hospices Civils de Lyon, Centre de Ressources et de Compétences de la Mucoviscidose (Adulte), GH Sud, Service de Médecine Interne, 69495 Pierre-Bénite, France; 7 Univ Lyon, Université Claude Bernard Lyon 1, RESHAPE, INSERM U1290, 69008 Lyon, France; 8 Univ Lyon, Université Claude Bernard Lyon 1, ISPB—Faculté de Pharmacie de Lyon, 69008 Lyon, France

**Keywords:** cystic fibrosis, therapeutic drug monitoring, tobramycin, pharmacokinetics, model-informed precision dosing

## Abstract

Therapeutic drug monitoring (TDM) of tobramycin is widely performed in patients with cystic fibrosis (CF), but little is known about the value of model-informed precision dosing (MIPD) in this setting. We aim at reporting our experience with tobramycin MIPD in adult patients with CF. We analyzed data from adult patients with CF who received IV tobramycin and had model-guided TDM during the first year of implementation of MIPD. The predictive performance of a pharmacokinetic (PK) model was assessed. Observed maximal (Cmax) and minimal (Cmin) concentrations after initial dosing were compared with target values. We compared the initial doses and adjusted doses after model-based TDM, as well as renal function at the beginning and end of therapy. A total of 78 tobramycin courses were administered in 61 patients. After initial dosing set by physicians (mean, 9.2 ± 1.4 mg/kg), 68.8% of patients did not achieve the target Cmax ≥ 30 mg/L. The PK model fit the data very well, with a median absolute percentage error of 4.9%. MIPD was associated with a significant increase in tobramycin doses (*p* < 0.001) without significant change in renal function. Model-based dose suggestions were wellaccepted by the physicians and the expected target attainment for Cmax was 83%. To conclude, the implementation of MIPD was effective in changing prescribing practice and was not associated with nephrotoxic events in adult patients with CF.

## 1. Introduction

Pulmonary exacerbations (PE) are common infectious complications in patients with cystic fibrosis (CF) [1]. Non-fermenting gram-negative bacilli are the most retrieved agents in PE, especially *Pseudomonas aeruginosa* [2]. The recommended therapy of PE in patients with CF is the association of a beta-lactam (e.g., ceftazidime, or piperacillin/tazobactam) with an aminoglycoside, both administered by intravenous (IV) route [3]. Tobramycin is the most widely used aminoglycoside agent in this setting due to its activity against *P. aeruginosa*. As PE are recurrent, repeated courses of such antibiotic therapy are necessary in patients with CF.

Aminoglycosides have a narrow therapeutic margin, and their antibacterial effect is concentration dependent [4]. Overexposure has been associated with ototoxicity and nephrotoxicity [5]. For initial dosing, recommended doses of tobramycin range from 10–15 mg/kg/day in CF patients [6]. However, dose individualization is required because of the narrow therapeutic margin and large interindividual pharmacokinetic (PK) variability. Therapeutic drug monitoring (TDM) has been recommended in this context to adjust the dosage and optimize the efficacy and safety in each patient [7,8].

However, TDM alone may not be an optimal approach for dose individualization of tobramycin. Traditional TDM only provides information on drug exposure to the physicians. Then, physicians have to interpret this information and use it adequately to adjust the dosage in order to achieve the pharmacokinetics/pharmacodynamics (PK/PD) target, and this process remains empirical. Limited information exists on how well TDM information is used to adjust aminoglycoside dosage in clinical routine. Model-informed precision dosing (MIPD) is an emerging approach that consists of using a pharmacokinetic model to interpret TDM results and compute the individual dose necessary to achieve a PK/PD target [9,10]. A Bayesian approach implemented in PK software is usually carried out to estimate individual PK parameter values based on both population information (population PK model) and individual information (dosing history, covariates such as renal function or body weight, and measured drug concentrations).

The objective of this study was to report our experience and first results of the implementation of an MIPD service for dosage individualization of tobramycin following TDM in adult patients with CF.

## 2. Materials and Methods

### 2.1. Tobramycin Local Dosing and Monitoring Practice

In our adult CF centre, PE were treated at home in most patients, with antibiotics administered as an outpatient parenteral antimicrobial therapy (OPAT). The initial dose was set by physicians based on previous courses. IV tobramycin was administered once daily for 14 days. A beta-lactam was commonly co-prescribed (e.g., ceftazidime or piperacillin/tazobactam). The preparation of tobramycin infusion bags was centralized in our central hospital pharmacy (Pharmacie Centrale des Hospices Civils de Lyon) and the preparations were then transported to patients’ homes. Tobramycin infusion duration was set at 30 min but sometimes varied slightly in each patient. TDM was performed once during therapy, on the third or fourth day, with sampling of trough concentration (Cmin) being just before the next infusion and the peak (Cmax) being 30 min after the end of the infusion. Again, true sampling times may have varied slightly but those were precisely recorded by nurses. Blood samples were transported to the hospital pharmacology laboratory that performed tobramycin assay. Prior to MIPD implementation, the TDM results were interpreted, and the tobramycin dosages were adjusted empirically by physicians alone.

Physicians used tobramycin concentration targets recommended by the French National Drug Agency; these were Cmax of 30–40 mg/L and Cmin < 0.5 mg/L at 24 h [11]. The Cmax target is based on a Cmax/MIC (minimal inhibitory concentration) target of 8 to 10 and a putative maximal MIC of 4 mg/L, which is the tobramycin breakpoint from the Clinical and Laboratory Standards Institute (CLSI) for susceptible strains of *P. aeruginosa* [12].

**Figure 1:**
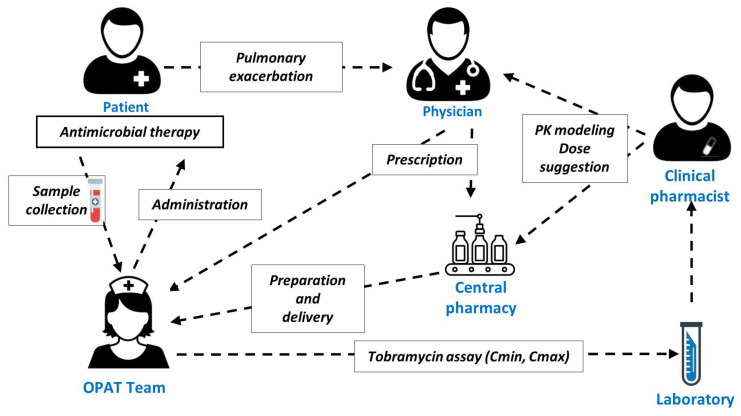
Organization of tobramycin therapy in our adult CF center. Abbreviations: Cmin, trough concentration; Cmax, maximal concentration; OPAT, outpatient parenteral antimicrobial therapy; PK, pharmacokinetics.

MIPD of tobramycin was implemented routinely in January 2021. Clinical pharmacists interpreted the TDM results with PK software (see below) and provided a dosage recommendation to physicians. The central pharmacy responsible for infusion bag preparation was also informed and could adjust the dose to be administered, if necessary. The organization of tobramycin therapy after implementation of MIPD is depicted in Figure 1.

For the initial dose, the physicians prescribed the same dose as the post-TDM dose of the previous course. As no patient exhibited severe renal impairment at baseline (see results), once daily dosing was applied, in accordance with guidelines [13,14].

### 2.2. Data Collection

We performed a retrospective analysis of data from all adult patients with CF who received IV tobramycin and had TDM from January 2021 to January 2022, after MIPD implementation. As this was a non-interventional study with TDM performed as part of routine patient care, no informed consent nor ethics approval was required, in accordance with the French law on biomedical research [15].

The data collected included blood sampling times, drug administration times, intravenous infusion duration, and measured drug concentrations, as well as patient characteristics including sex, age, body weight, serum creatinine, and creatinine clearance (CL_CR_, estimated with the Cockcroft–Gault equation) at tobramycin therapy onset and at the end of therapy. We also collected data on PK modelling (see below), including the predicted tobramycin concentrations and PK parameter values (central volume of distribution and clearance) as well as data on dosage adjustments including the tobramycin initial dose, the dose suggested by clinical pharmacists after TDM and PK modelling, and the dose set by physicians after this recommendation.

Concentrations of tobramycin were measured by using an automated immunoturbidimetry assay (PETIA). The lower limit of quantification was 0.2 mg/L. Intra- and inter-day repeatability expressed as coefficients of variation were less than 4%. The method was validated according to our national quality insurance program.

### 2.3. MIPD and PK Data Analysis

The TDM results were analysed by using a Bayesian PK modelling approach. We used the BestDose^TM^ software to perform Bayesian fitting of the PK model and estimation of individual PK parameters (e.g., clearance and volume of distribution) in each patient on all TDM occasions [16]. The measured Cmin < 0.2 mg/L were set at 0.1 mg/L (half the lower limit of quantification) in the Bayesian PK modeling.

Bayesian estimation of PK parameters was based on a nonparametric two-compartment population model previously developed and validated by our group in children and adolescents with CF [17]. The population distributions of PK parameters were used as prior in the Bayesian estimation. Extrapolation to adult patients was theoretically possible since this model includes the influence of physiological variables that are scalable; the tobramycin elimination rate constant is linearly correlated with creatinine clearance (in mL/min) and the central volume of distribution is expressed in L/kg. The equations of the covariate-parameter relationships are as follows:

V1 = Vs. × BW(1)

where V1 is the tobramycin central volume of distribution (in L), Vs. is the central volume of distribution in L/kg, and BW is the body weight in kg. The symbol “S” indicates that Vs. is the slope parameter in the regression of V1 versus BW.

Ke = K_I_ + K_S_ × CL_CR_ (2)

where Ke is the tobramycin elimination rate constant (in h^−1^), CL_CR_ is the creatinine clearance estimated by the Cockcroft-Gault equation (in mL/min), K_S_ is the slope parameter in the regression of Ke versus CL_CR_, and K_I_ is the non-renal component of elimination (intercept parameter in the regression of Ke versus CL_CR_).

Good predictive performance of the model in adult patients has been recently confirmed in another dataset from adult patients [18].

Once the model had been fit to the data and provided acceptable results, it was used to simulate a future once-daily dosing regimen. The dosage was computed to achieve the recommended targets cited above: Cmax of 30 to 40 mg/L and Cmin < 0.5 mg/L. Because real infusion and sampling times for Cmax could differ from the standard, the model was used to calculate Cmax 30 min after the end of a 30 min infusion (Cmax_mod_), and this value was considered in the target attainment and dose adjustment.

The PK report sent to physicians included three recommended dosages for achieving the lower, mid-value, and upper bounds of the target interval (30, 35, and 40 mg/L, respectively). Due to the tobramycin presentations available in France, the dose suggestions were rounded to the next 25 mg dose.

### 2.4. Statistical Analysis

To assess the goodness-of-fit of the PK model, the individual predicted concentrations were compared with the observed concentrations. Target attainment after initial dosing was assessed by the proportion of Cmax, Cmax_mod,_ and Cmin within the target range. We evaluated the effects of MIDP by comparing the initial dose to the dose finally selected by the physicians with the Wilcoxon signed-rank test for paired samples. To assess the effect of dose changes on renal function, baseline serum creatinine and creatinine clearance were compared with values at the end of tobramycin therapy with the same test. A *p*-value less than or equal to 0.05 was considered as significant in all tests. An increase of 50% of serum creatinine from baseline was considered as a marker of acute kidney injury (AKI).

## 3. Results

During the study period of one year, 78 tobramycin courses were administered in 61 patients. One patient was excluded because his age was ≤18 years. The characteristics of the population are presented in Table 1 and the PK results in Table 2. The tobramycin median initial dose (9.2 mg/kg) was slightly lower than the recommended dose of 10 mg/kg, and only 27% of the initial doses were within the range 10–15 mg/kg. Renal function was normal in most patients. Mild renal impairment (creatinine clearance between 60 and 90 mL/min) was observed in eighteen (23%) patients and moderate renal impairment (creatinine clearance between 30 and 60 mL/min) was observed in one (1.3%) patient. No patients had severe renal impairment.

**Figure 2:**
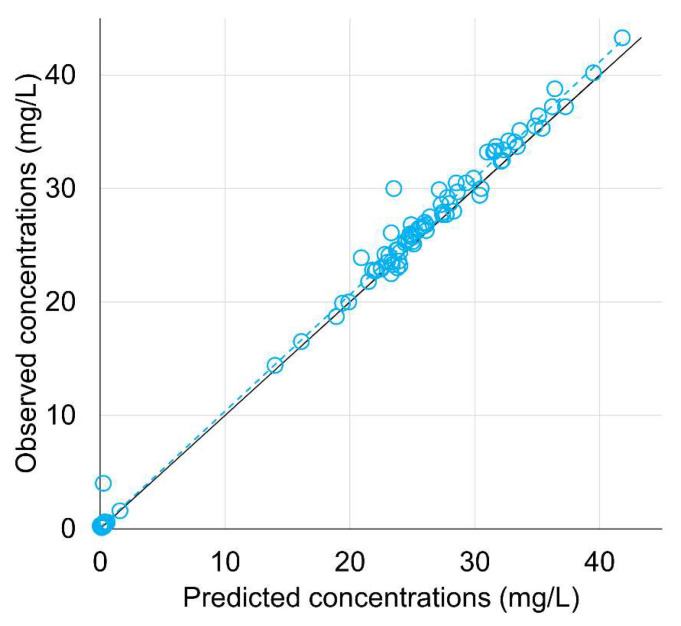
Observed concentrations of tobramycin versus individual model predictions. Blue circles represent observation/prediction pairs. The dashed blue line is the linear regression line. The solid line is the line of identity (*y* = *x*).

The tobramycin PK model fit the data very well, as shown in Figure 2. The model predictions were highly correlated with the observations (R^2^ > 0.99). The predictive performance was very good with a median (interquartile range) prediction error of −0.11 mg/L (−0.8; 0) and a median absolute percentage error of 4.9% (2.5%; 24.4%).

After initial dosing, 68.8% of patients had a Cmax_mod_ value below the lower bound of the target interval (30 mg/L). Overexposure (Cmax > 40 mg/L) was observed in only one patient who received an initial dose of 9.2 mg/kg. Regarding Cmin, 88.3% of patients had values < 0.5 mg/L and 67.9% of patients had a Cmin value lower than 0.2 mg/L.

**Figure 3:**
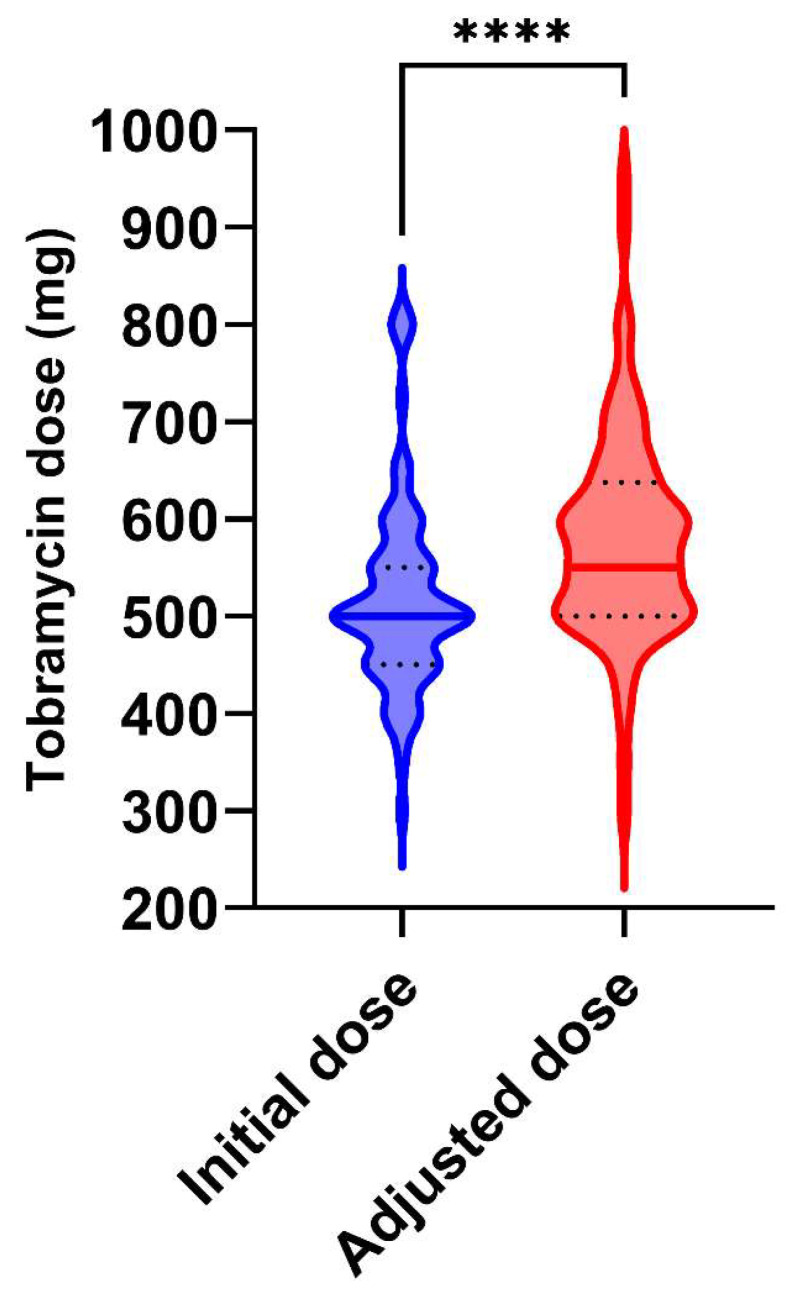
Violin plots of tobramycin doses before and after dose adjustment. The central solid line is the median. The black dotted lines are the quartiles (25th and 75th percentiles). Symbol **** indicates *p* < 0.0001.

Table 3 summarizes the dose changes after model-guided TDM. Overall, the dose was unchanged in twenty-eight cases (36.4%), while it was increased in forty-six cases (59.7%) and decreased in three cases (3.9%). After the physicians’ decision, 64 patients (83.1%) were expected to have tobramycin concentrations between 30 and 40 mg/L, with doses ranging from 6.1 to 14.7 mg/kg (median: 10.2 mg/kg). The comparison of the initial and adjusted doses of tobramycin is shown in Figure 3. While the initial doses were evenly distributed around a median of 500 mg, the model-guided dose adjustment resulted in a higher median dose of 550 mg (*p* < 0.001) and a larger variability, reflecting the individual dosage requirements. At the end of the antibiotic course, neither a new course nor prolongation of the cure were required for any patient, which suggests treatment efficacy.

**Figure 4:**
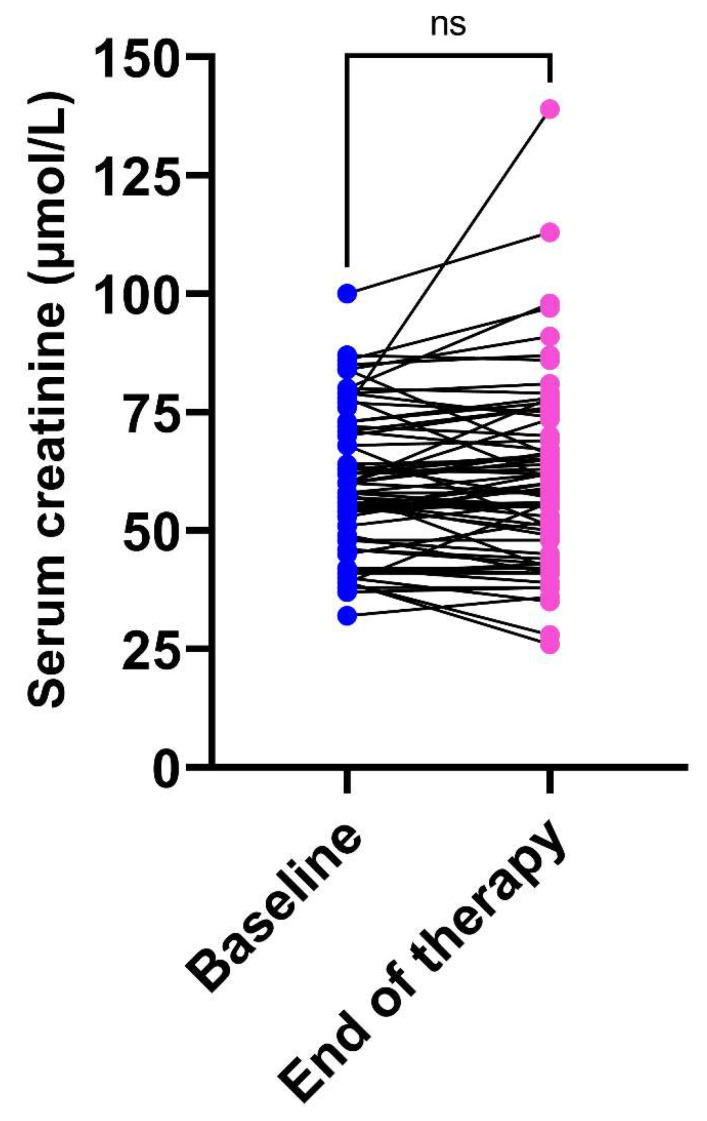
Individual changes in serum creatinine during tobramycin therapy (*n* = 69). Abbreviation: ns, non-significant.

Regarding the renal function under therapy, there was no statistical difference between serum creatinine at baseline and at the end of therapy (*p* = 0.697). Individual changes in serum creatinine during therapy are shown in Figure 4. Of note, serum creatinine at the end of therapy was not available in eight patients. Only one patient (1.3%) showed an increase in serum creatinine greater than 50% from baseline, which was considered as a marker of acute kidney injury. This patient received an initial dose of 500 mg (8.2 mg/kg), which was increased to 650 mg (10.7 mg/kg) after model-guided TDM showed a measured Cmax of 26.8 mg/L (Cmax_mod_ = 24.6 mg/L). His serum creatinine increased during therapy from 76 to 139 µM.

## 4. Discussion

The appropriate treatment of *Pseudomonas aeruginosa* pulmonary exacerbations in CF patients has a major impact on patients’ quality of life. In this context, patients are expected to receive numerous courses of antibiotics with potential side effects associated. TDM of antibiotics has been supported in numerous reports and guidelines for optimizing the efficacy and safety of antimicrobial therapy, especially in special populations [19,20]. However, TDM alone may not be an optimal approach if the interpretation of results and dose adjustments remain empirical. This was reported more than 20 years ago by van Lent-Evers et al., who showed that active model-guided TDM of aminoglycosides was associated with better concentration target attainment and clinical outcomes than standard TDM with empirical dosing [21]. In a recent study from our group, we have shown that empirical dose adjustment performed by physicians after TDM of tobramycin in CF adult patients often failed to adequately modify tobramycin dosages for achieving the Cmax target [18]. In patients with observed tobramycin Cmax lower than the target, empirical dose increases were too low for achieving the expected values in most patients. This previous study and the present one confirm that model-guided TDM, now described as MIPD, is more effective than conventional TDM in adjusting the dosage of tobramycin in goal-oriented therapy.

Considering that the initial dose represented the local dosing practice before implementation of MIPD in our cohort, our results showed that most patients (74%) received an initial tobramycin dose lower than 10 mg/kg. As a result, 68% of patients exhibited an estimated Cmax lower than the target of 30 mg/L. The MIPD approach was effective in changing clinician dosing practice and lead to a higher tobramycin dose. Those higher doses were expected to result in better target attainment. However, this could not be evaluated in the present study because TDM was not repeated during PE therapy and a second cure within the same year was rarely administered.

In our experience, the MIPD service provided by pharmacists was well accepted by physicians, with an acceptance rate of dose suggestion of 83.1%.

Physicians declined a model-based dose adjustment in *n* = 13 patients (16.9%) and decide to keep the initial dose despite under- or over-exposure. The main reasons were concerns about patient frailty, renal function, or pregnancy.

Importantly, there was no significant change in renal function with the dose increases suggested by MIPD. Only one patient out of sixty-nine (1.4%) showed an increase in serum creatinine over 50%. This toxicity rate is similar to the literature data [22,23]. This suggests that higher doses of once-daily tobramycin are probably safe, although further research is required to confirm this result.

Regarding the PK model and the software used for MIPD, we confirmed that a tobramycin PK model developed in children and adolescent with CF was adequate for fitting concentration in adults. Other studies reported successful model extrapolation in populations different from the one used in model building [24,25]. A strength of the BestDose^TM^ software is that it includes a hybrid-fit option, which basically consists of increasing PK parameter ranges and reducing prior information to identify parameter values out of the bounds of the original prior distribution [26,27]. This option was used in some patients of our cohort when the standard fit was not adequate (data not shown).

Another strength of model-guided TDM is the ability to interpret drug concentrations when infusion and sampling times deviate from the standards. This is especially important in the interpretation of Cmax because a small variation in sampling time can result in quite a large change in measured concentrations. In our study, the median sampling time for Cmax was 30 min, but significant deviations occurred in some patients (min, 4 min; max, 69 min).

This study has some limitations. Data were collected in routine clinical practice, so errors may have occurred in their reporting. We consider the estimated creatinine clearance as the covariate influencing tobramycin elimination, in accordance with the original model developed in children [17]. However, it would be interesting to evaluate other indices of renal function in future studies, such as other creatinine-based equations (e.g., CKD-EPI, Lund-Malmö), cystatin C-based equations, or serum cystatin C as performed elsewhere [28]. The achievement of the PK/PD objectives after dose adjustment was not evaluated because a second TDM during therapy was not routine practice in our center. The Cmax/MIC target was not based on measured MIC but on the CLSI breakpoint. Lower exposure may be adequate in the case of pathogens with a lower MIC. We did not consider a clinical endpoint of the tobramycin therapy, such as forced expiratory volume in one second (FEV) [29], because it was not performed routinely at home in our cohort. Only nephrotoxicity was considered in the safety assessment. Further clinical research with multiple efficacy and safety endpoints and a longer follow-up are necessary to confirm the value of tobramycin MIPD in patients with CF.

## 5. Conclusions

To conclude, in our experience in adult patients with CF, underexposure to tobramycin was frequently observed after empirical initial dosing. Implementation of an MIPD service provided by pharmacists to physicians resulted in significant increases in tobramycin doses without significant impact on renal function. Dosage adjustments were well-accepted by physicians. Further clinical evaluation is required to evaluate other potential benefits. TDM alone is not sufficient for precision dosing of antibiotics. MIPD appears to be a promising step forward.

## Figures and Tables

**Table 1:** Patient characteristics.

Variable	Value
Number of patients (number of women/men)	77 (53/24)
Age (years)	32.4 ± 10
Body weight (kg)	57.5 ± 12.3
Body mass index (kg/m^2^)	20.9 ± 4.0
Tobramycin initial dose (mg)	518.2 ± 98.2
Tobramycin initial dose (mg/kg)	9.16 ± 1.42
Tobramycin initial dose between 10–15 mg/kg	27.3%
Baseline CL_CR_ (mL/min)	112.7 ± 28.4
CL_CR_ at the end of therapy (mL/min) ^a^	112.6 ± 33.3
Difference between final and initial CL_CR_ (mL/min)	0.62 ± 17.8
Serum creatinine increase ≥50% from baseline	1.3 % (*n* = 1)

^a^ serum creatinine at the end of therapy was not available for eight patients. Values are given as mean ± standard deviation unless otherwise stated. Abbreviations: CL_CR_, creatinine clearance.

**Table 2:** Pharmacokinetic results.

Variable	Value
Infusion time (min)	35.9 ± 7.4
Cmax post-infusion sampling time (min)	32.1 ± 8.9
Measured Cmax (mg/L)	27.8 ± 5.4
Estimated Cmax (mg/L)	27.0 ± 5.2
Cmax_mod_ (mg/L)	28.2 ± 4.3
Cmax_mod_ < 30 mg/L	68.8%
Cmax_mod_ between 30 and 40 mg/L	28.9%
Cmax_mod_ > 40 mg/L (%)	1.3%
Measured Cmin at 24 h (mg/L)	0.25 ± 0.48
Estimated Cmin at 24 h (mg/L)	0.22 ± 0.19
Cmin at 24 h < 0.5 mg/L (%)	88.3%

Abbreviations: Cmax, maximal concentration; Cmax_mod_, concentration estimated 30 min after the end of a 30 min infusion; Cmin, trough concentration. Values are given as mean ± standard deviation unless otherwise stated.

**Table 3:** Dose changes after model-guided TDM according to measured concentrations.

Estimated Cmax Value (mg/L)	No Dose Change (%)	Dose Increase (Median, min–max) in mg	Dose Decrease (Median, min–max) in mg	Accepted Model-Based Dose Suggestion Targeting				Total
				30 mg/L (%)	35 mg/L (%)	Between 30 and 35 mg/L	40 mg/L (%)	
<30	12 (21.4%)	44 (100, 25–200)	0	24 (42.9%)	6 (10.7%)	14 (25%)	0 (0%)	56 (72.7%)
30–35	11 (78.6%)	2 (87.5, 75–100)	1 (75)	2 (14.3%)	1 (7.1%)	0 (0%)	0 (0%)	14 (18.2%)
35–40	4 (66.7%)	0	2 (112.5, 25–200)	1 (16.7%)	1 (16.7%)	0 (0%)	0 (0%)	6 (7.8%)
>40	1 (100%)	0	0	0 (0%)	0 (0%)	0 (0%)	0 (0%)	1 (1.3%)
Total	28 (36.4%)	46 (59.7%)	3 (3.9%)	27 (35.1%)	8 (10.4%)	14 (18.2%)	0 (0%)	77 (100%)

## Data Availability

Data are available upon demand.
